# Effect of acacia honey on cultured rabbit corneal keratocytes

**DOI:** 10.1186/1471-2121-15-19

**Published:** 2014-05-26

**Authors:** Choy Ker-Woon, Norzana Abd Ghafar, Chua Kien Hui, Yasmin Anum Mohd Yusof

**Affiliations:** 1 Department of Anatomy, Faculty of Medicine, Universiti Kebangsaan Malaysia, Jalan Raja Muda Abdul Aziz, 50300 Kuala Lumpur, Malaysia; 2 Medical Molecular Biology Institute, Universiti Kebangsaan Malaysia, Kuala Lumpur, Malaysia; 3 Department of Physiology, Faculty of Medicine, Universiti Kebangsaan Malaysia, Kuala Lumpur, Malaysia; 4 Department of Biochemistry, Faculty of Medicine, Universiti Kebangsaan Malaysia, Kuala Lumpur, Malaysia

**Keywords:** Acacia honey, Corneal keratocytes, Proliferation

## Abstract

**Background:**

Acacia honey is a natural product which has proven to have therapeutic effects on skin wound healing, but its potential healing effects in corneal wound healing have not been studied. This study aimed to explore the effects of Acacia honey (AH) on corneal keratocytes morphology, proliferative capacity, cell cycle, gene and protein analyses. Keratocytes from the corneal stroma of six New Zealand white rabbits were isolated and cultured until passage 1. The optimal dose of AH in the basal medium (FD) and medium containing serum (FDS) for keratocytes proliferation was identified using MTT assay. The morphological changes, gene and protein expressions of aldehyde dehydrogenase (ALDH), marker for quiescent keratocytes and vimentin, marker for fibroblasts were detected using q-RTPCR and immunocytochemistry respectively. Flowcytometry was performed to evaluate the cell cycle analysis of corneal keratocytes.

**Results:**

Cultured keratocytes supplemented with AH showed no morphological changes compared to control. Keratocytes cultured in FD and FDS media supplemented with 0.025% AH showed optimal proliferative potential compared with FD and FDS media, respectively. Gene expressions of ADLH and vimentin were increased in keratocytes cultured with AH enriched media. All proteins were expressed in keratocytes cultured in all media in accordance to the gene expression findings. No chromosomal changes were detected in keratocytes in AH enriched media.

**Conclusion:**

Corneal keratocytes cultured in media supplemented with 0.025% AH showed an increase in proliferative capacity while retaining their morphology, gene and protein expressions with normal cell cycle. The results of the present study show promising role of AH role in accelerating the initial stage of corneal wound healing.

## Background

Cornea is an avascular and transparent tissue located at the anterior aspect of the eye. It consists of five layers, i.e. the epithelium, Bowman’s membrane, stroma, Descemet’s membrane and endothelium. The corneal stromal comprises 90% of total corneal thickness and made up of precise organization of the collagen fibres such as collagen type I and type V and extracellular matrix (ECM) which contributes to the bulk of the structural framework and mechanical stability of the cornea. Stromal collagen fibrils are bounded by proteoglycans such as keratan or chondroitin sulphate, which control the hydration [1]. Keratocytes are the major cell type populated in the stroma and mainly involved in maintaining the extracellular matrix environment. These cells contain corneal ‘crystallins’, which is responsible for reducing scattering of light from the keratocytes and maintaining transparency [2].

Eye injuries frequently caused by chemical, thermal, infection, trauma or surgical procedure such as keratectomy [3,4]. These injuries may involve only the epithelium, for example, in corneal abrasion [5] or may extend deep into the stroma such as corneal ulceration [6,7]. The conventional treatment for corneal injury involves topical administration of antibiotic or antifungal eye drop to combat secondary infection [8]. Unfortunately, few side effects have been reported such as antibiotic resistance due to prolong use and development of keratitis [9,10]. Common preservatives used to maintain sterility of the eye drop such as benzalkonium ammonium chloride can cause destruction of the corneal epithelium, tear film instability and ultimately delayed wound healing [11-13]. All preservatives, even at very low concentrations, appeared to be cytotoxic for ocular cells and ultimately leads to apoptosis and free radical production [14,15].

Over the past few years, there has been a constant effort by many researchers to overcome the side effects of the conventional treatment. Hence, there is a need for alternative agent such as natural products, which may act as an adjuvant to the current treatment. Honey is a natural product which is well known for its medicinal properties. The main constituents of honey are sugars, water, trace elements and some chemical compounds such as phenolic acids and flavonoids [16]. Honey possesses a wide range of medicinal properties such as antibacterial, antioxidants, anti-inflammatory, antithrombotic and anti-allergic activities [17-19].

Acacia honey is produced by cultured bees, *Apis mellifera,* which collect the nectar from *Acacia mangium* trees and flowers [20,21]. It has been reported to promote skin wound healing caused by burn injury [22]. However, its effect on ophthalmological disorder has not been elucidated. Skin fibroblasts and corneal keratocytes are both mesenchymal derived cells during development [23,24]. Hence, we postulate that AH might have the same healing properties on the cornea. To the best of our knowledge, this may be the first study of its kind, which reported the proliferative activity of AH on in vitro corneal keratocytes.

## Methods

The present study was performed according to the ethical consideration laid down by the Universiti Kebangsaan Malaysia Animal Ethics Committee (permit number: UKMAEC Approval Number FP/ANAT/2012/NORZANA/18-JANUARY/419-JANUARY-2011-DECEMBER-2013-AR-CAT2).

### AH sample

AH was purchased from Ministry of Agriculture and gamma irradiated at 25 kGy [18] at Ministry of Science, Technology and Innovation, Malaysia. AH was then stored at room temperature.

### Rabbit corneal keratocytes isolation and cell culture

Six New Zealand white rabbits’ eyes were obtained from the local animal slaughter house, and the corneal tissues were harvested and processed using the techniques reported earlier [25]. Harvested cornea was incubated in Dispase solution 2 mg/ml (Sigma-Aldrich, USA) at 4°C for 18 hours to detach the epithelium from the stroma. Corneal stroma was rinsed with PBS (pH 7.2, Gibco Invitrogen, USA) and each stromal tissue was cut into half. Each piece was digested with 0.3% collagenase type I, incubated at 37°C with intermittent gentle shaking until all the connective tissues were digested. The isolated keratocytes in the suspension were centrifuged at 500 × g for 10 minutes. The resultant pellet was washed with phosphate buffered solution (PBS, pH 7.2, Gibco Invitrogen, USA) to remove any residual enzyme and suspended in the PBS for total cell quantification with haemocytometer (Weber Scientific Int, Ltd. Middlx, England). Cell viability was determined by trypan blue dye (Gibco Invitrogen, USA) exclusion test. Viable keratocytes were seeded in six well-plates (BD Falcon, Franklin Lakes, NJ) with seeding density of 5 × 10^3^ cell/cm^2^ in a complete medium consisting of Ham’s F-12:Dulbecco’s Modified Eagle’s Medium (Gibco), 10% foetal bovine serum (FBS; Gibco), 1% of antibiotic and antimitotic (Gibco), 1% of 50 μg/ml ascorbic acid (Sigma, St. Louis, USA). All cultures were maintained in 5% CO_2_ incubator (Jouan, Duguay Trouin, SH) at 37°C under 95% humidity and the media were changed every three days. Upon 80% confluence, the primary culture (P0) was trypsinized using 0.125% trypsin-EDTA (Gibco) and subculture until passage 1 (P1). The morphological features were examined everyday using an inverted phase contrast microscope (Carl Zeiss, Germany).

### MTT assay

MTT (3-[4, 5-dimethylthiazolyl-2]-2, 5-diphenyltetrazolium bromide; Sigma-Aldrich) assay was used for quantitative evaluation of AH on corneal keratocytes’ viability and proliferation. Corneal keratocytes from passage 1 were used and seeded in a 96-well cell culture plate (Cellstar, Greiner Bio-one, Germany) for 24 hours with the seeding density of 5 × 10^3^ cells/ cm^2^. Then the medium was changed to serum-free medium (FD) and medium containing serum (FDS) supplemented with different concentration of AH from 0% to 3.125% using dilution factor of two. Corneal keratocytes were incubated at 37°C in a humidified incubator 5% CO_2_ for 48 hours. MTT assay was performed by adding 10 μl MTT solution into each well and incubated for another four hours in the dark. The formazan crystals that formed by living cells were solubilized by 100 μl Dimethylsulfoxide (DMSO) at each well, and the absorbance was measured at 570 nm by ELISA reader. The total viable cell number was directly proportional to the level of absorbance produced by the purple formazan precipitate. AH concentration which provided the highest cell proliferation was chosen as the optimal dose for subsequent tests, i.e. gene, protein and cell cycle analyses. The optimal dose of AH was identified as 0.025% concentration. Hydrogen peroxide (H_2_O_2_) at the concentration of 1.56nM was chosen as positive control, which produced IC_50_ for corneal keratocytes according to the previous pilot study.

### Total RNA extraction and gene expression analyses

Keratocytes from passage 1 were cultured in four different media; A) serum-free medium (FD), B) FD with 0.025% AH, C) medium containing serum (FDS) and D) FDS with 0.025% AH. Total RNA from keratocytes was isolated using TRI Reagent (Molecular Research Centre, Cincinnati, USA) according to the manufacturer’s protocol. Chloroform was added into the TRI Reagent homogenate to separate the colourless aqueous contained total RNA. Isopropanol and polyacryl carrier (Molecular Research Centre) was added to each extraction to precipitate the total RNA. The extracted RNA pellet was washed with 75% ethanol and air dried before dissolving it in Rnase and Dnase free distilled water (Invitrogen, Carlsbad, USA). Complementary DNA was synthesised from 100 ng of Total RNA with SuperScript™ III First-Strand Synthesis SuperMix Reverse Transcriptase (Invitrogen, Carlsbad, USA) according to the manufacturer’s protocol. In brief, primer annealing was performed at 23°C for 10 minutes, reverse transcription at 50°C for 60 minutes and termination of reaction at 85°C for 5 minutes. The expression of aldehyde dehydrogenase (ALDH) and vimentin were evaluated by two-step reverse transcriptase-polymerase chain reaction (Invitrogen, Carlsbad, USA). Expression of glyceraldehyde- 3-phosphate dehydrogenase (GAPDH) gene was used as housekeeping gene. The primers (sense and antisense) used for quantitative PCR reaction were designed, based on the sequences published in GenBank using Primer-3 software as shown in Table 1. The two-step RT-PCR reaction was performed using SYBR Green as the indicator in Bio-Rad iCycler (Bio-Rad, USA). Each reaction mixture consisted of 12.5 μl of iQ SYBR Supermix, forward and reverse primers (1 μl each), deionised water and 1 μl of cDNA template. The reaction conditions were cycle 1: 95°C for 3 minutes (1 ×), cycle 2: Step 1 95°C for 10-second and Step 2 61°C for 30-second (40 ×), followed by melting curve analysis. The specificity and the PCR product size were confirmed by 2% agarose gel electrophoresis.

**Table 1:** Primers used in qRT-PCR gene expression analyses

**Gene**	**GenBank accession number**	**Primer sequence 5′- 3′**	**PCR product size (bp)**
GAPDH	NM_001082253	F: caa cga att tgg cta cag ca	186
		R: aaa ctg tga aga ggg gca ga	
ALDH	AY508694	F: gsg tgg cat gat tca gtg agc	186
		R: gag tag tcg tcc cct ctt gga	
Vimentin	AY465353.1	F: tgc agg aag aga ttg cct tt	117
		R: tga ggt cag gct tgg aga ca	

### Immunocytochemistry

Keratocytes in FD and FDS with and without supplementation of 0.025% AH were fixed in 4% paraformaldehyde for 15 minutes at 4°C. Cells were then incubated with primary antibodies for 30 minutes. Primary antibodies used were anti-ALDH (1:200, Dako) and anti-vimentin (1:200, Dako). Nuclei were stained with haematoxylin (Sigma). Positive stained cells exhibited brownish precipitate in the cytoplasm using confocal laser scanning microscopy (LSM-510, Zeiss). The same keratocytes under same culture medium without primary antibody staining served as negative control.

### Cell cycle analysis

The cell cycle properties of corneal keratocytes were analysed in all culture media with or without supplementation of 0.025% AH. Passage 1 corneal keratocytes was seeded at density of 1.3 × 10^5^ cells/cm^2^ in the T75 culture flask (Cellstar, Greiner Bio-one, Germany). After 24 hours, the medium was changed to four different media, which were FD medium, FD with 0.025% AH, FDS medium and FDS with 0.025% AH. After 48 hours incubation at 37°C in a humidified incubator 5% CO_2_, cells were trypsinized with trypsin and centrifuged. Cells were stained with Cycle Test Plus DNA Reagent Kit (Becton Dickinson). Cell cycle distribution was analysed by flowcytometry (Becton Dickinson, FACS Canto II). Propidium iodide was excited at 50 nm. Total of 15,000 events in each sample was acquired. Using BD FASCSDiva software, the percentages of cells at different phases of the cell cycle were determined.

### Statistical analysis

Data was tested for statistical significance using the statistical package for Social Sciences (SPSS) version 20. Values were expressed as mean ± standard error of mean (SEM) and were analysed using Student’s t-test and One-way Analysis of Variance (ANOVA). A p-value of less than 0.05 was considered significant.

## Results

### Cell viability and proliferation assay

**Figure 1:**
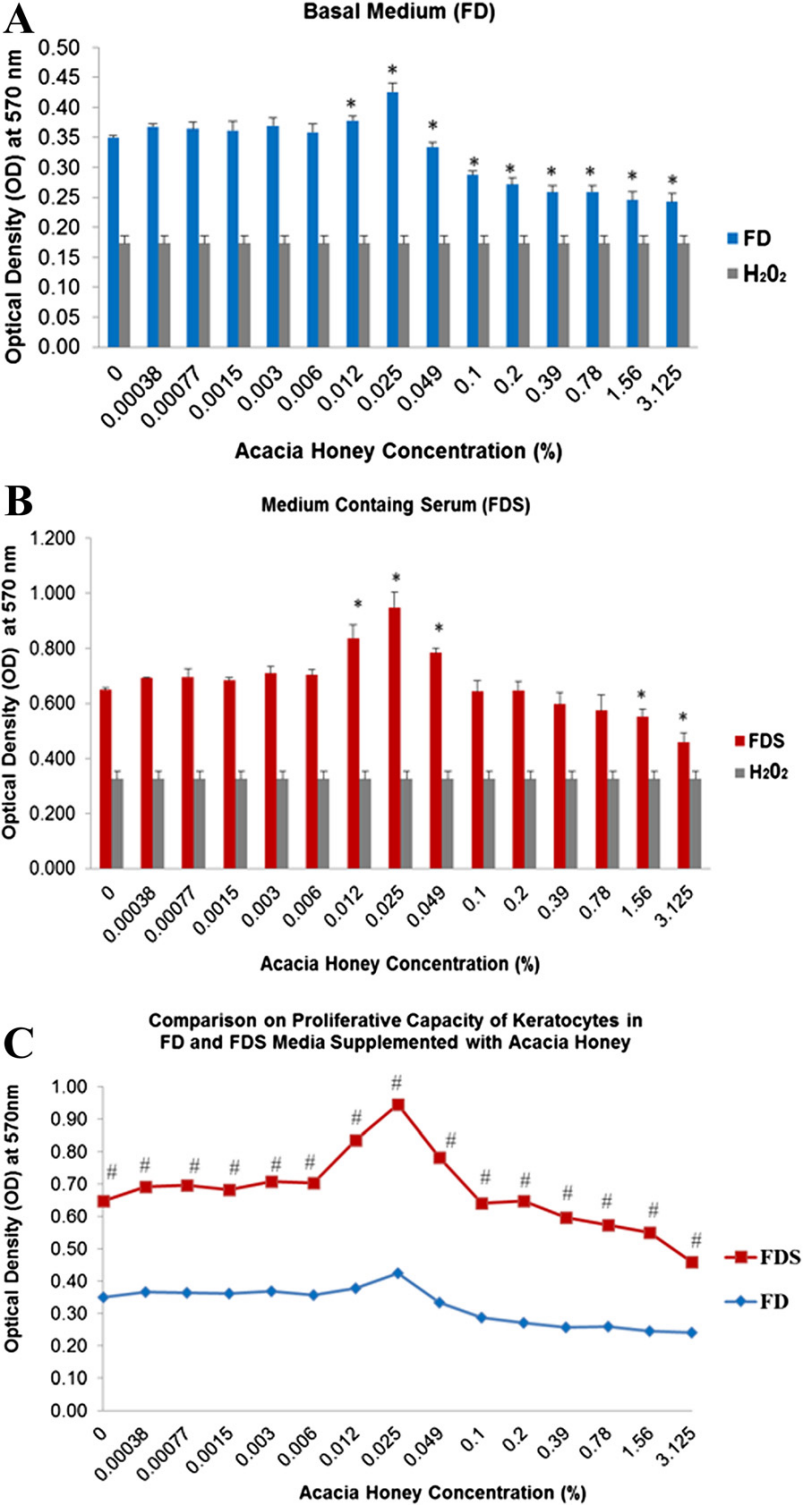
**Viability of corneal keratocytes cultured in two different media. A)** serum-free medium (FD) supplemented with AH ranging from 0% to 3.125%; **B)** medium containing serum (FDS) supplemented with AH ranging from 0% to 3.125%; **C)** comparison of the cell viability between the FD and FDS supplemented with different concentrations of AH. Significant difference (p < 0.05) between the same medium was marked with (*) while (#) denotes significant difference (p < 0.05) between different media. Values were expressed as mean ± SEM, n = 6.

Corneal keratocytes cultured in the FD medium supplemented with low concentrations of AH from 0.00038% to 0.025% exhibited increase in proliferative capacity compared to the FD medium alone. Corneal keratocytes cultured in the FD medium supplemented by 0.012% AH (p = 0.024) and 0.025% AH (p = 0.003) showed a significant increase in the proliferative capacity (p < 0.05) when compared to the FD medium alone (Figure 1A). Subsequent concentrations of AH from 0.049% to 3.125% showed a significant decrease in the pattern of cell proliferation compared to control. Corneal keratocytes cultured in the FD medium supplemented with 0.025% AH showed the highest proliferative capacity.

Corneal keratocytes cultured in the FDS medium showed higher proliferative potential at 0.012% (p = 0.01), 0.025% (p = 0.003) and 0.049% AH (p = 0.00) compared to the FDS alone (Figure 1B). Corneal keratocytes showed a reduction in proliferation in FDS medium containing AH concentration ranging from 0.1% to 3.125% AH compared to the control group. Proliferative capacity was significantly lower in the corneal keratocytes cultured in 1.56% (p = 0.02) and 3.125% AH (p = 0.002).

Corneal keratocytes cultured in FDS medium exhibited higher cell proliferative capacity compared to the FD medium, with or without supplementation of AH (Figure 1C). The differences were significant among cells cultured in the FDS and FD media at the concentration ranged from 0% to 3.125% AH (p < 0.05).

### Cell morphological study

**Figure 2:**
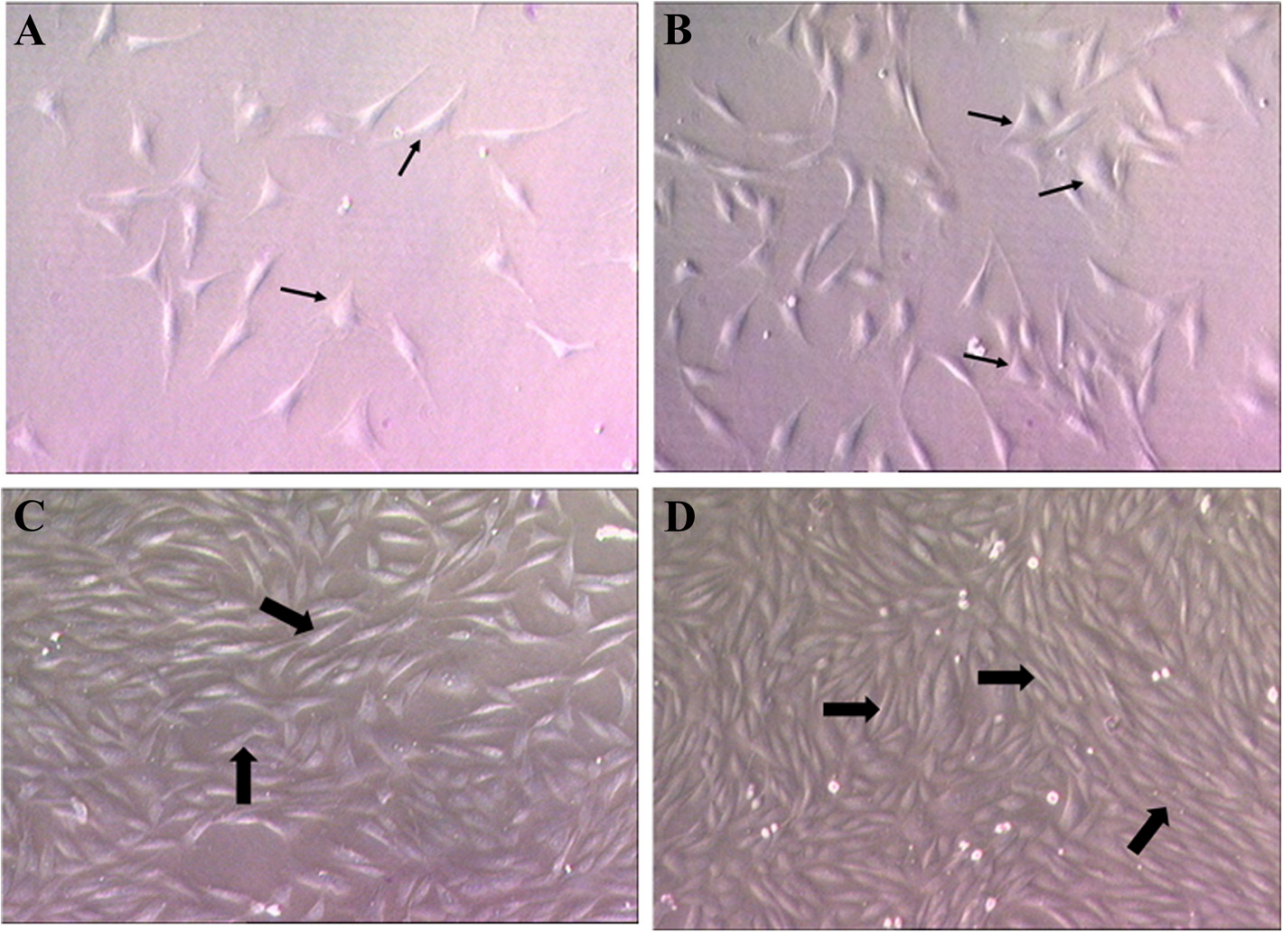
**Phase contrast micrographs of the corneal keratocytes at passage 1, 48 hours after culturing in four different media. A)** basal medium, FD; **B)** FD with 0.025% AH; **C)** medium containing serum, FDS; **D)** FDS with 0.025% AH. Thin arrow (→) showed fibroblast with dendritic morphology with multiple extended cytoplasmic processes. Thick arrow (➡) showed fusiform-shaped cells. Supplementation of 0.025% AH promotes higher cell density in both FD and FDS media (50X).

Keratocytes cultured in the absence of serum exhibited a dendritic morphology with multiple extended cytoplasmic processes (Figure 2A & B). Corneal keratocytes cultured in the presence of serum exhibited fusiform-shaped cells with retraction of dendritic processes (Figure 2C & D). This morphology was most apparent in the FDS medium supplemented with 0.025% AH (Figure 2D). Cultured keratocytes supplemented with 0.025% AH in the FD (Figure 2B) and FDS (Figure 2D) media showed higher cell density compared to the FD and FDS media respectively (Figure 2A & C).

### Gene expression analysis

**Figure 3:**
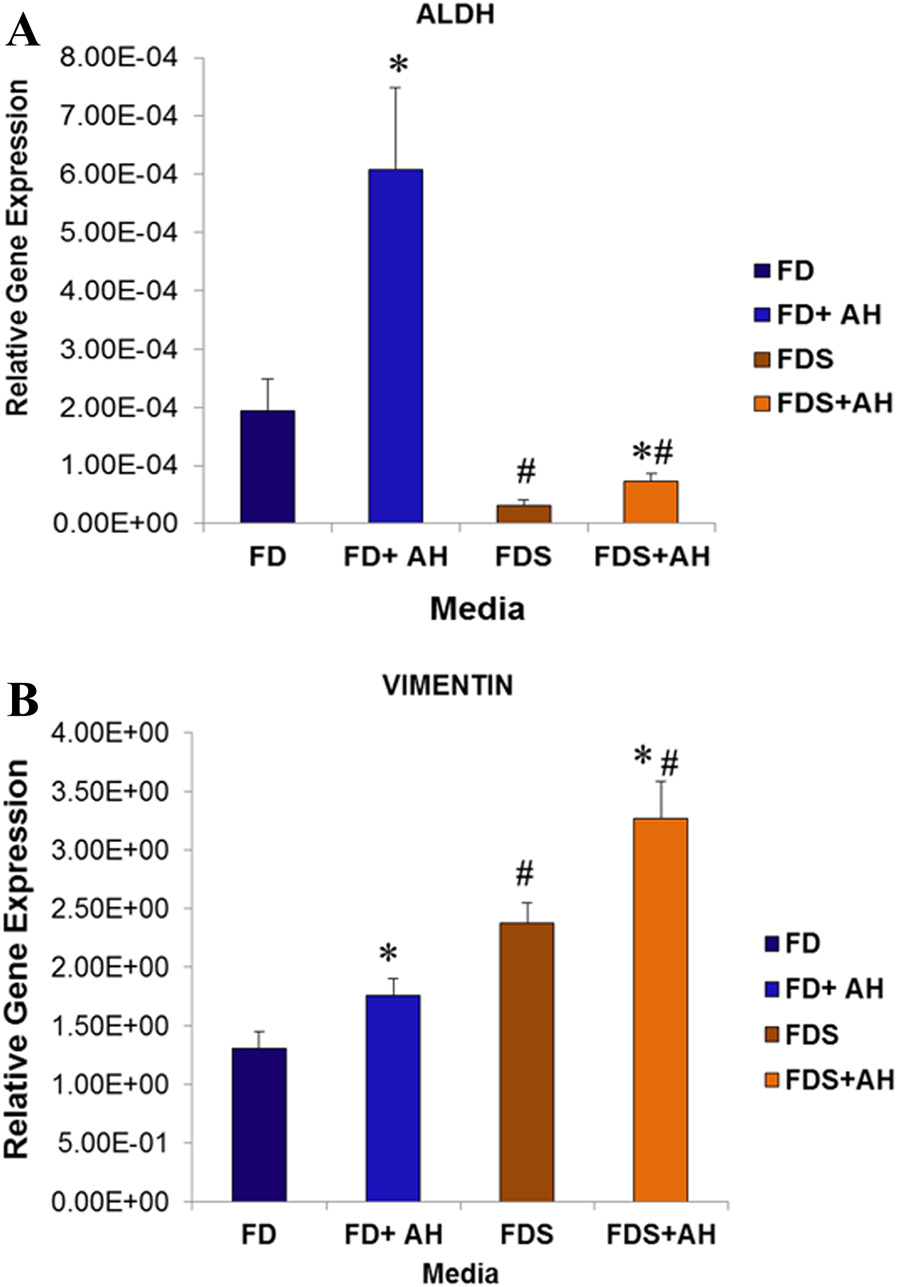
**Quantitative gene expression of cultured corneal keratocytes.** The expression values for **A)** ALDH and **B)** Vimentin relative to the gene expression values of GAPDH as the internal control. (*) denotes significant difference (p < 0.05) in the same group. (#) denotes significant difference (p < 0.05) between groups. Values were expressed as mean ± SEM, n = 6.

**Figure 4:**
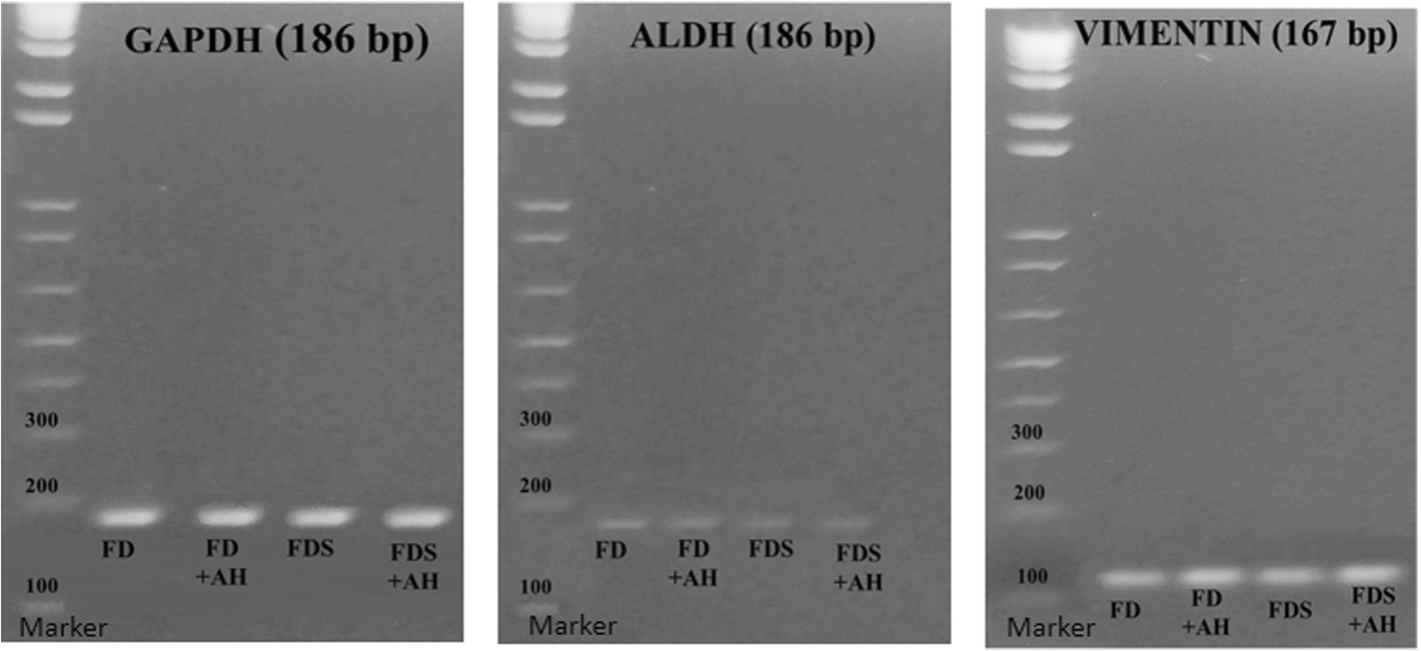
Gel electrophoresis of cultured rabbit corneal keratocytes phenotypes with GAPDH gene as internal control.

Keratocytes cultured with 0.025% AH in the FD and FDS media showed a significant increased in the mRNA expression of aldehyde dehydrogenase (ALDH) compared to the FD and FDS media respectively. Expression of ALDH was higher in the FD group compared to the FDS group (p < 0.05) either with or without supplementation of AH (Figure 3A). Keratocytes supplemented with 0.025% AH in FD and FDS media showed a significant increase in the mRNA expression of vimentin compared to the FD and FDS media, respectively (Figure 3B). Gel electrophoresis of ALDH and vimentin genes demonstrated the specific size product of corneal keratocytes in all culture media (Figure 4).

### Protein expression analysis

**Figure 5:**
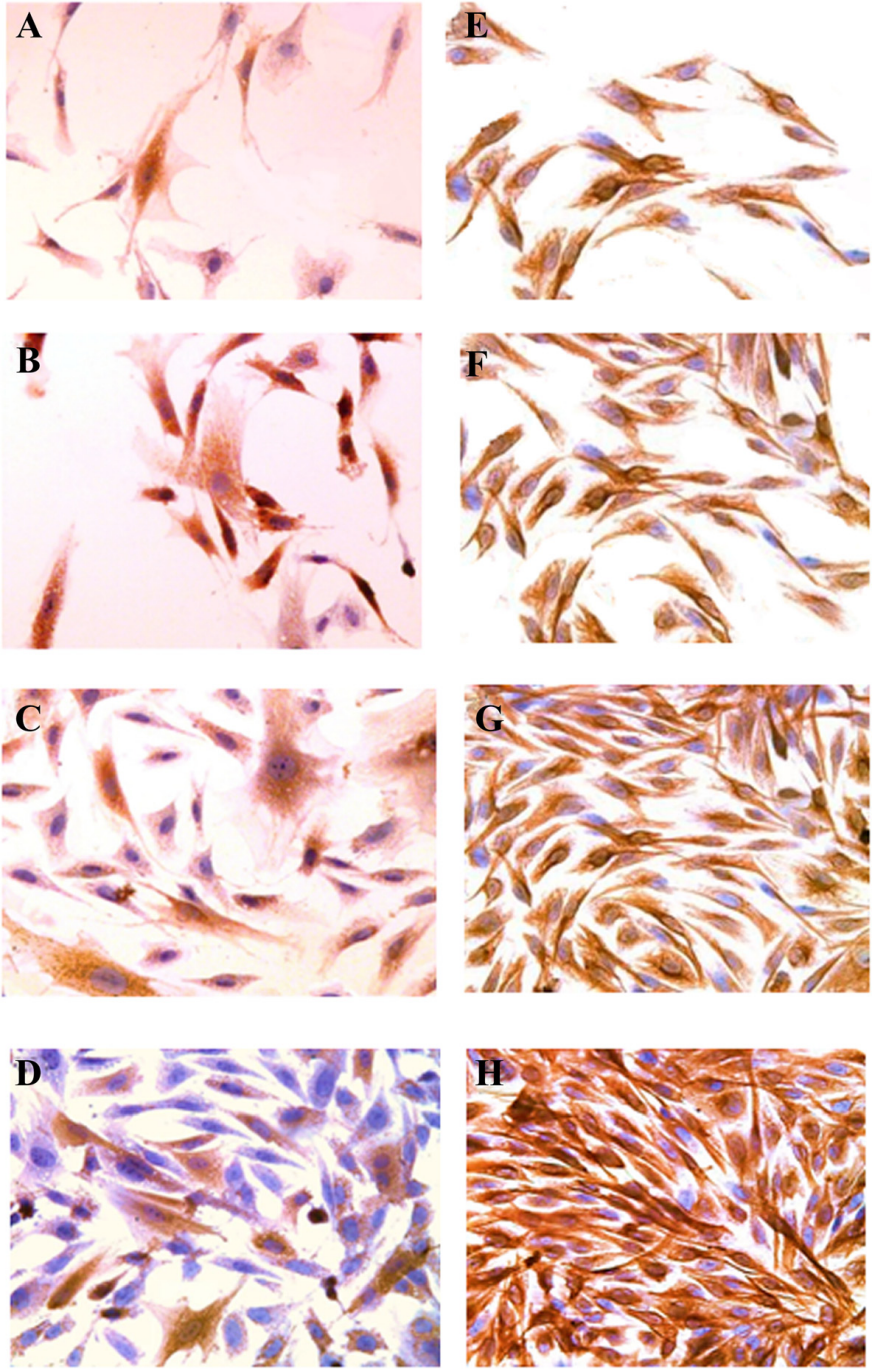
**Immunocytochemistry of corneal keratocytes.** Expression of ALDH: **A)** basal medium, FD; **B)** FD with 0.025% AH; **C)** medium containing serum, FDS; **D)** FDS with 0.025% AH. Expression of vimentin: **E)** basal medium, FD; **F)** FD with 0.025% AH; **G)** medium containing serum, FDS; **H)** FDS with 0.025% AH (200X).

The ALDH protein was expressed higher in the FD group (Figure 5A) compared to FDS group (Figure 5C). The FD with the supplementation of 0.025% AH (Figure 5B) exhibited higher expression of ALDH protein compared to the FDS supplemented AH group (Figure 5D). However, expression of vimentin protein was higher in the FDS group (Figure 5G) compared to the FD group (Figure 5E). The FDS supplemented with 0.025% AH (Figure 5H) expressed higher vimentin protein compared to FD supplemented AH group (Figure 5F).

### Cell cycle analysis

**Figure 6:**
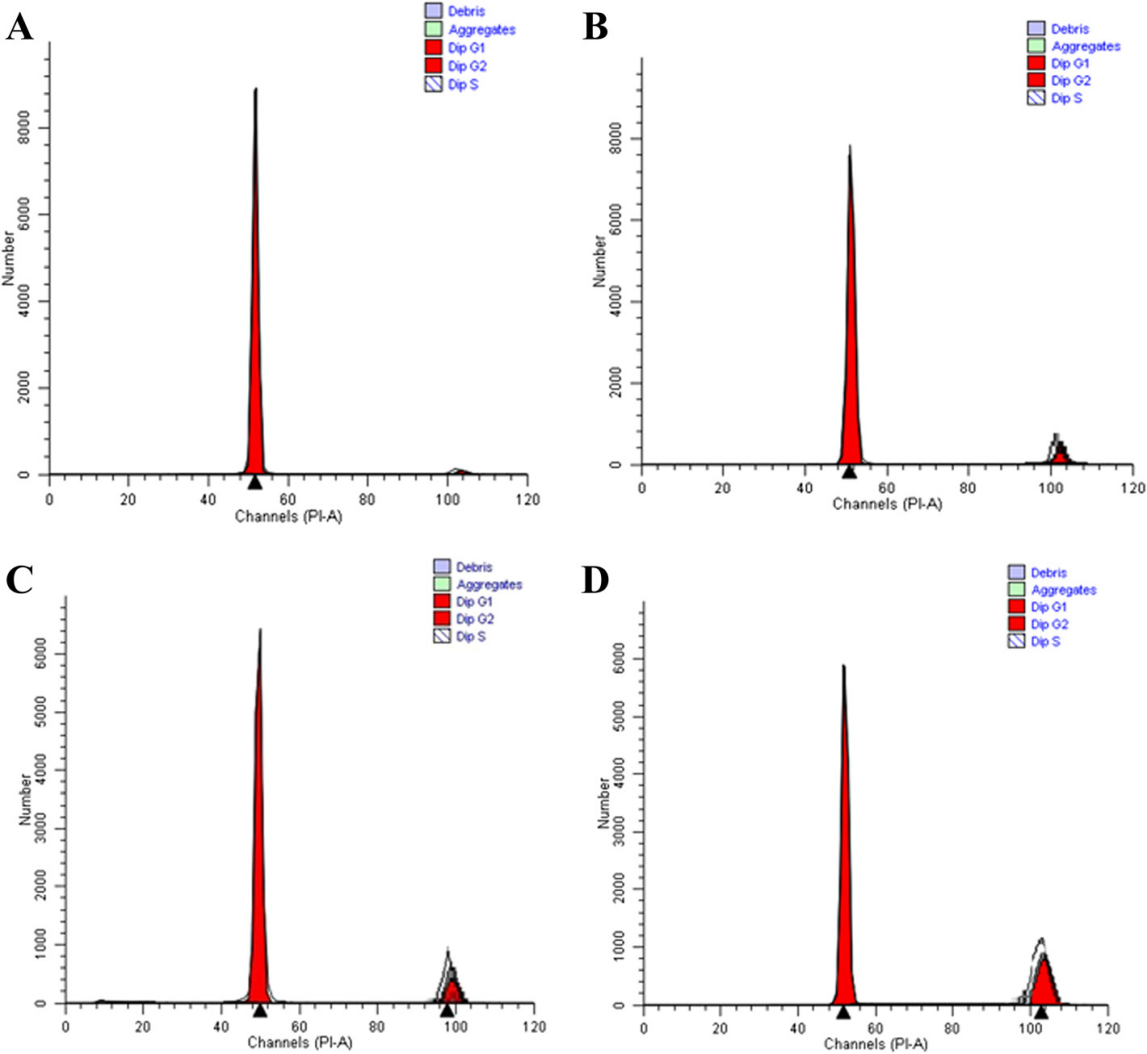
**DNA histogram.** Percentage of G0-G1, S, G2 and M phases generated from corneal keratocytes cultured in four different media: **A)** basal medium, FD; **B)** FD with 0.025% AH; **C)** medium containing serum, FDS; **D)** FDS with 0.025% AH. X-axis represents the relative fluorescence intensity proportional to DNA content.

DNA histograms in all culture media showed the same cell cycle pattern indicating an absence of aneuploidy or tetraploidy (Figure 6). All cultured corneal keratocytes were in the diploid state. Keratocytes cultured in FD (Figure 6A) and FDS (Figure 6C) media showed a higher percentage of G0-G1 phase in the absence of AH. Keratocytes cultured in FD (Figure 6B) and FDS (Figure 6D) supplemented with 0.025% AH showed a significant increase in percentage of S-phase and G2-M phase compared to control (Table 2).

**Table 2:** DNA content (in percentage) of keratocytes cultured in four different media

**Medium**	**Cell cycle phases**		
	**G0/G1**	**G2/M**	**S**
FD	90.96 ± 0.3343	3.51 ± 0.1108	4.52 ± 0.8050
FD + 0.025% AH	89.63 ± 0.8101*	4.11 ± 0.1404*	5.25 ± 0.2231*
FDS	87.77 ± 0.4043	5.98 ± 0.2063	6.17 ± 0.1797
FDS + 0.025% AH	81.66 ± 0.6368**	7.94 ± 0.1907**	8.88 ± 0.2306**

(*) denotes significant difference (p < 0.05) between FD supplemented with AH compared to FD medium alone. (**) denotes significant difference (p < 0.05) between FDS supplemented with AH compared to FDS medium alone (**p < 0.05) at different cell cycle stages. Value were expressed as mean ± S.E.M.

## Discussion

Suitable phenotypic conversion is essential for cell regenerative therapy and tissue engineering of the corneal stromal [26]. Keratocytes cultured in the absence of serum exhibited a broad, stellate and dendritic morphology with numerous cell processes stretching from the cell body in all directions [27]. In the presence of serum, keratocytes lost their quiescence and exhibited spindle-shaped morphology with greater organelle contents and size of the cells. These cells were reported to possess numerous nucleoli and were deficient in cytoplasmic granules [28], indicating the transformation from keratocytes to fibroblast [29]. These findings are similar to the present study.

Serum contains growth factors, hormones, amino acid, protein, mineral, fatty acids and lipid [30] which promote the transition of quiescent keratocytes to fibroblasts and myofibroblasts [31]. This transition is important in acquiring a sufficient number of cells for wound healing. Morphological changes of keratocytes in the present study are comparable to stromal cells during in vivo wound healing [32]. It has been reported that cultured keratocytes exhibited heterogeneous population, which consists of discrete keratocytes phenotypes such as quiescent keratocytes, fibroblasts and myofibroblasts [33].

The process of cell proliferation in corneal wound healing is an active and energy-consuming process [34]. The major constituents of AH are sugar, which consists mainly of fructose and glucose [20,35]. The sugars in honey possess properties such as viscosity, granulation and provides energy from glycolysis [35,36]. In this study, cell proliferative assay showed corneal keratocytes proliferation increased significantly in FD and FDS media supplemented with 0.025% AH. Glucose provides energy, which is vital during proliferation in the initial stage of corneal wound healing. The results of the present study showed that 0.025% AH acts synergistically with serum containing medium in enhancing the proliferative capacity of corneal keratocytes. Interestingly, past researchers reported that low concentration (0.0195%) of Tualang honey was able to potentiate the proliferation of human osteoblast cell line (CRL 1543) and the human fibroblast cell line in the presence of foetal bovine serum in the culture media [37]. Another study showed higher concentration (2.4%) of Tualang honey caused cytotoxicity and apoptosis to human breast and cervical cancer cell lines [38]. Hence, honey exhibits an optimal proliferative capacity at lower concentration compared to that of higher concentration.

The phenotypical changes of corneal keratocytes can be distinguished by the expression of specific corneal marker, aldehyde dehydrogenase (ALDH) for quiescent keratocytes and vimentin for fibroblasts. ALDH, a group of water-soluble proteins contain corneal crystallins for corneal transparency. ALDH minimises fluctuations in the refractive index within the cytoplasm of the cells and matches the refractive index to the surrounding extracellular matrix [2]. In the present study, ALDH expression was higher in the FD group compared to FDS group. These findings were similar to an earlier study which evaluated the difference in mRNA expression of ALDH isoezymes in human corneal keratocytes and repair fibroblasts [39]. ALDH was expressed abundantly in the keratocytes phenotype in serum-free medium [40]. The results of the present study revealed that the expression of vimentin was up-regulated in FDS group compared to FD group. Presence of serum was reported to induce transformation from keratocytes to the repair phenotype, i.e. fibroblast and it is accompanied by loss of corneal crystallins [29,41,42]. The supplementation of AH in culture media increased both the expression of ALDH and vimentin compared to the controls, respectively. This indicates that AH could promote corneal transparency and synthesis of ECM during wound healing. Immunocytochemistry results for ALDH and vimentin proteins were also in accordance to the qRT-PCR results.

Cell cycle analysis is used to quantify the cells’ proportion during cell division at various phases; G0, G1, S-phase (synthesis) and G2-M (mitosis). It is essential that the proliferating keratocytes do not possess aneuploidy state that may lead to generation of malignant phenotype. In this study, additional AH to the culture media increases the proliferation of keratocytes (increase in S phase and G2-M phase), whilst maintaining the normal diploid state of the cells. These findings were in conformity with the MTT assay results.

## Conclusion

In conclusion, AH at a concentration of 0.025% significantly promotes corneal keratocytes proliferation, suggesting its potential effect on the regeneration of corneal keratocytes during the initial stage of wound healing. Addition of AH promotes the emergence of the repair phenotype, i.e. fibroblasts, which is evidenced in the gene and protein expressions. Addition of AH maintains normal cell cycle and did not induce any chromosomal abnormalities. The present study on the proliferative capacity of AH opens the door for future research in innovating and commercialising Acacia honey-based eye drop for therapeutic purposes.

## Competing interests

The authors declare that they have no competing interests.

## Authors’ contributions

NAG designed the research, analyzed data and involved in manuscript editing. CKW performed the experiments, analyzed data and drafted the manuscript. CKH and YAMH involved in discussion, analyzed data and manuscript editing. All authors read and approved the final manuscript.
